# Oxidant/Antioxidant Equilibrium and Neurotransmitter Levels in Camelids Used for Circus Activities: A Preliminary Study

**DOI:** 10.3390/vetsci12060570

**Published:** 2025-06-10

**Authors:** Raffaella Cocco, Federica Arrigo, Sara Sechi, Maria Rizzo, Giuseppe Piccione, Francesca Arfuso

**Affiliations:** 1 Department of Veterinary Medicine, University of Sassari, 07100 Sassari, Italy; rafco@uniss.it (R.C.); sarasechilavoro@tiscali.it (S.S.); 2 Department of Veterinary Sciences, University of Messina, 98168 Messina, Italy; rizzom@unime.it (M.R.); gpiccione@unime.it (G.P.); farfuso@unime.it (F.A.)

**Keywords:** circus, serotonin, oxidative stress, camelids, dopamine, noradrenaline

## Abstract

The conditions of animals in captivity have long been a cause for concern, and for that reason should be carefully assessed. In circus activities, animals are used for different purposes, but their needs are different due to their physiological peculiarities. The aim of the present study was to investigate the emotional state and the oxidant/antioxidant equilibrium in dromedaries, camels, and llamas subjected to circus management. Blood samples were taken from five specimens of three different species in order to assess the serum concentrations of noradrenaline, dopamine, serotonin, and the plasma levels of reactive oxygen metabolites (d-Roms) and the animals’ biological antioxidant potential (BAP). The results showed higher levels of d-Roms in dromedaries and llamas than camels and higher concentrations of BAP in camels than other species. Finally, dromedaries showed a higher dopamine concentration than llamas. This preliminary study suggested that some of the species investigated here are more influenced by the circus environment, especially on a relational level. Although the results collected in this study are preliminary and need to be supported by further, more comprehensive investigations from the point of view of welfare assessment, it could be hypothesized that the emotional state and the oxidant/antioxidant balance is very important to assess in an environment such as the circus.

## Simple Summary

This study examined the welfare of dromedaries, camels, and llamas under circus management by analyzing their emotional states and oxidant/antioxidant balance. Blood samples from five animals per species were tested for neurotransmitters and oxidative stress markers. Dromedaries and llamas showed higher oxidative stress (d-Roms) than camels, while camels had greater antioxidant capacity (BAP). Dromedaries also had more dopamine than llamas, suggesting greater emotional reactivity. These preliminary findings highlight species-specific differences in how circus environments may affect animals’ well-being and emphasize the need for further research.

## 1. Introduction

There are five main physical/functional and mental domains that can be assessed to understand the condition of animals in captivity [1]. These include nutrition, environment, physical health, behavior, and lastly, affective states. Places such as aquariums, zoos and circuses represent cause for concern in terms of these issues. This has led to public demand for stricter legislation and new methods of welfare assessment [2,3].

Due to their unique characteristics, camelids are among the most commonly used animals in zoos and circuses. Species such as *Camelus dromedarius*, *Camelus bactrianus*, and *Lama glama* are generally docile and are often used in obstacle courses or by circus performers for acrobatics routines [4]. The decision to use these animals is likely due to their interesting adaptive and physiological characteristics. Physical characteristics such as the structural features of red blood cells contribute to their high resistance to osmotic lysis, decreased deformability, and high affinity for oxygen. This enables them to withstand certain stress situations such as hot environments and a possible lack of water and food [5]. Although different camelids have different requirements, poor feeding, transport, or housing in cages are simply a few examples of how their welfare can be affected in places like circuses. Indeed, the difference between the animals’ various diets, the frequency of feeding, and the quantity and quality of food are clear limitations [6]. Additionally, transporting the animals from one city to another can mean that the animals are forced to stay in containers, suffering movement, vibration, and noise from the vehicles. This can cause the activation of catatonic pathways, dehydration, fatigue, immunosuppression, and sleep disturbances [7].

Animals relocated from captivity to the circus must cope with the new stimuli and the new environment, possibly establishing new coping strategies [8]. Exposure to different stimuli results in the activation of hormonal systems such as the sympathetic adrenal system and the hypothalamus–pituitary–adrenal (HPA) axis. These collaborate in the ‘fight-or-flight’ system with the production of adrenalin and noradrenalin, increased heart rate, heart pressure, and an increased state of alertness. The HPA axis acts via a cascade system involving the mobilization of energy reserves to support the ‘fight-or-flight’ reaction [9,10]. The sympathetic system is responsible for the adrenergic response and its activation also leads to the secretion of dopamine and serotonin. On a daily basis, a variety of stimuli can activate either the serotonin or noradrenalin pathway; possible production of serotonin implies docility and friendliness; on the contrary, activation of the serotonin pathway indicates stressful and anxious situations [11,12,13]. The constant release of stress hormones can lead to negative health outcomes [13] and when testing the body’s psychological defenses, investigating the oxidative stress response represents a useful tool, since it gives us an idea of the balance between oxidants and antioxidants [14,15].

To the authors’ knowledge, no previous studies have specifically assessed sympatho-adrenal responses and plasma neurotransmitter levels in camelids kept in captivity. Studying these parameters could be considered important, as it contributes to understanding the emotional state of the animal, particularly with respect to animal welfare and behavior. Norepinephrine, in fact, is involved in the mechanisms of stress, fear, and the sympathetic response. Dopamine, on the other hand, plays a fundamental role in motor control and the reward system. Serotonin is localized in humor management [16,17]. Evaluation of the antioxidant balance, together with the above, can give an insight into the redox state of the animal. In light of these considerations, it is necessary to consider that, to the authors’ knowledge, no further studies investigating these parameters in circus animals exist in the literature, so the aim of the present preliminary study was to investigate the basal plasma levels of some substances considered biomarkers of well-being in domestic animals, and specifically related to the animal’s mental state, such as noradrenaline, dopamine, and serotonin in dromedaries, camels, and llamas in several Italian circuses. In addition, reactive oxygen metabolites (d-Roms) and biological antioxidant potential (BAP), which denote the oxidant/antioxidant balance of organisms, were assessed.

## 2. Materials and Methods

### 2.1. Animals and Study Design

The study design has been approved by the Animal Welfare Body and Experimentation of the University of Sassari (Prot. n. 4537 of 21 January 2025). The animal husbandry and experimentation protocols were reviewed and approved in accordance with the standards recommended by the Guide for the Care and Use of Laboratory Animals and Directive 2010/63/EU for animal experiments. For the study, 15 circus animals of different species (i.e., camels, dromedaries, and llamas) were used with the prior permission of their owners. The three species are listed in Table 1 along with their age, mean body masses, sample sizes, and box sizes. The blood samples were collected at rest during a normal working day in the circus. The animals were housed in semi-shaded open barns under natural environmental conditions (mean temperature 10.4 ± 3.3 °C; mean relative humidity 79%). All animals were fed a concentrate feed mixture consisting of 65% yellow corn, 20% wheat bran, 10% soybean meal, and 5% cottonseed meal, in addition to alfalfa hay and rice straw. Water was available ad libitum. All animals were subjected to biochemistry analyses (Table 2), and all were clinically healthy and free from internal and external parasites.

### 2.2. Blood Sampling and Analysis

Blood samples were obtained via jugular venipuncture, and the samples were collected in 8 mL vacutainer tubes containing a clot activator and 8 mL lithium heparin tubes (Terumo Co., Tokyo, Japan). All samples were taken in the boxes at rest (7:00 a.m.). Immediately after collection, blood samples were placed in refrigerated bags and transported to the laboratory for the analysis within three hours following sampling.

The procedures and laboratory analyses were described by Cocco et al. [13].

### 2.3. Statistical Analysis

The Shapiro–Wilk test was used to assess normality. All data were normally distributed (*p* > 0.05). One-way analysis of variance (ANOVA) was used to investigate differences in the values of circulating dopamine, noradrenaline, serotonin, d-Roms, and BAP among dromedaries, camels, and llamas kept in captivity. When significant differences were found (*p* < 0.05), Bonferroni’s test was performed for post hoc analysis. The data were analyzed with the software Prism v. 9.00 (GraphPad Software Ltd., San Diego, CA, USA, 2020).

## 3. Results

**Figure 1:**
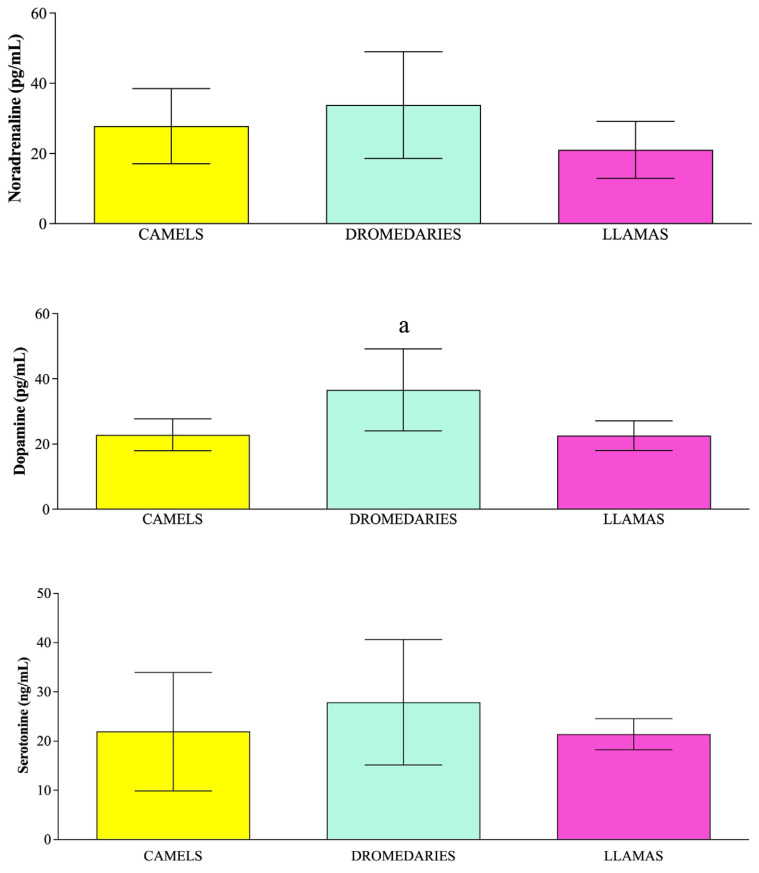
Mean values ± standard deviation (±SD) of serum concentration of noradrenaline, dopamine and serotonin measured in camels, dromedaries, and llamas, together with the relative statistical significance. Statistical significance (*p* < 0.05): a vs. llamas.

**Figure 2:**
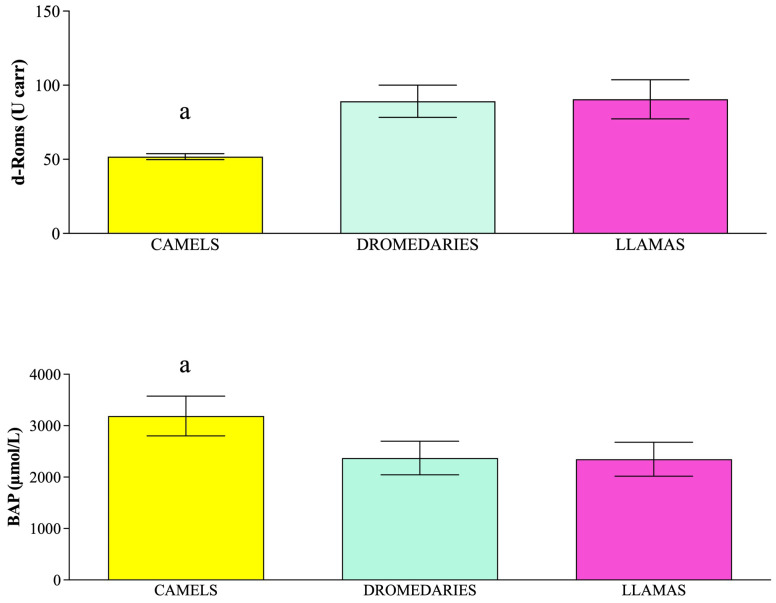
Mean values ± standard deviation (±SD) of plasma reactive oxygen metabolites (d-Roms) and biological antioxidant potential (BAP) values measured in camels, dromedaries and llamas together with the relative statistical significances. Statistical significance (*p* < 0.05): a vs. dromedaries and llamas.

Data were expressed as mean values ± standard deviations. According to the results gathered from statistical analysis, the concentration of parameters related to the emotional state (dopamine) and the oxidative balance (i.e., d-Roms and BAP) statistically changed among species (*p* < 0.05). Specifically, dromedaries showed higher dopamine concentrations than llamas (Figure 1, *p* < 0.05). Moreover, camels showed lower levels of d-Roms than dromedaries and llamas (Figure 2, *p* < 0.05) and higher BAP values compared to dromedaries and llamas (Figure 2, *p* < 0.05).

## 4. Discussion

The emotional state of animals in captivity is of considerable importance; wild animals have been used in entertainment for various purposes, and continue to be used in such a manner today. The situation of circuses is often complex, with so many animals being forced to perform difficult tricks, in some cases under constant threat of physical punishment. They are used as traveling actors, transported in cramped trucks and are kept locked in cages, and often their fate is to perform until the day they die. For this reason, the requirements of the different species are not considered compatible with the objectives of entertainment [17]. Living in stressful situations to which they are not accustomed, animals develop their own physiological systems to act as a buffer against adverse conditions in order to restore balance [13]. To the authors’ knowledge, there are no studies in the literature concerning the assessment of the mental state (noradrenaline, serotonin and dopamine) and oxidant/antioxidant balance (d-Roms and BAP) of camelids managed within a circus environment. In recent years, behavioral ecologists have used d-Roms (derivative reactive oxygen metabolites) to assess the oxidative stress that occurs when there is an excess of reactive oxygen species that exceeds the body’s ability to neutralize with antioxidants [17]; this can lead to cellular damage, death, or organ dysfunction. The results gathered in the current study showed higher concentrations of d-Roms in dromedaries and llamas than camels. This parameter, which can be used to assess pro-oxidant activity, is useful for understanding that there is higher production of free radicals in this species. The measurement of d-roms is considered important for assessing oxidative stress as a result of an imbalance between free radicals and antioxidants. This is due to the reaction of occasional stimuli but could influence health if it were to occur in the long term. The results could be related to certain situations that could certainly cause stress to animals that are not used to such scenarios. During a day at the circus, animals may be forced to take part in several performances, (which may be difficult and may be required to be repeated over time) and may be exposed to a lot of noise (due to music and the audience). They are often confined to small spaces and cannot interact socially. Finally, their diet is not well controlled, often not differentiated by species, and they are continuously transported between locations [18,19]. Exposure to events to which animals are not accustomed and which they have yet to learn to cope with could lead to a hormonal imbalance and an activation of the hypothalamic–pituitary–adrenal axis, which causes an endocrine imbalance that stimulates ROS production to a greater extent [20]. Compared to dromedaries and llamas, camels have evolved in more stable environments. In fact, the results of the present study show a lower concentration of d-roms and a higher concentration of BAP. The findings could be related to an increased efficiency in defense mechanisms against stress, with a greater ability to neutralize ROS through a particularly efficient antioxidant system. For this reason, certain events typical of the circus environment, such as movements, manipulations, and habitat transformations, can interrupt or interfere with social processes [20].

The results obtained showed significant differences (*p* < 0.05) in neurotransmitter levels among species. Dopamine is linked to the regulation of fatigue and endurance and it has been found that it can be determined at high concentrations during desert crossing. Therefore, what was observed in this study may be related to the ability of dromedaries to endure and respond to harsh conditions [20]. Dopamine concentrations may also be involved in social, motivation and reward regulation in circus activity [21,22]. In contrast, llamas seem not to be driven by survival needs. For that reason, their social relationships are less demanding, and we can thus explain the lower dopamine concentration compared to dromedaries [23,24,25].

## 5. Conclusions

According to the authors’ knowledge and findings, this is the preliminary study comparing the mental state and oxidative/antioxidant balance of different species used as circus animals. The results obtained could show that among the species used, camels respond to the stimuli of the circus environment with a higher concentration of BAP than other species to balance oxidative stress. Dromedaries seem to develop new resources to react positively to their new environment through increased dopamine production. Based on the preliminary results obtained, more attention should be paid to factors that may influence the emotional state of the circus environment. However, although the results collected in the present study may provide useful information on the emotional state of animals employed in circus work, they are not yet sufficient to draw definitive conclusions and need further confirmation and investigation. Therefore, further prospective studies analyzing a larger number of time points and animals and taking into account additional indicators of good mental and behavioral states could provide a more holistic view of the welfare state of animals maintained under circus management.

## Figures and Tables

**Table 1:** Signalment data for the five species enrolled in the study, with the number of subjects written in parenthesis.

Common Name	Scientific Name	Age (years)	Gender	Body Weight (kg)	Box Sizes (m)
Camel (5)	*Camelus bactrianus*	8–17	5 males	595 ± 21	2 × 5 × 5
Dromedary (5)	*Camelus dromedarius*	8–11	4 males, 1 female	789 ± 45	5 × 5 × 1.5
Llama (5)	*Lama glama*	2–9	2 males, 3 females	184 ± 21	4 × 4 × 1.5

**Table 2:** Mean ± standard deviation of serum biochemical parameters expressed in their common unit (total proteins—TP; albumin; urea; alkaline phosphatase—ALP; glucose—GLU; γ-glutamyltransferase—GGT; glutamate–oxaloacetate transaminase—GOT; glutamate pyruvate transaminase—GPT; total bilirubin; total cholesterol; triglycerides; calcium—Ca; phosphorus—P; creatine phosphokinase—CPK; creatinine) measured in camels—dromedaries, llamas and kept under circus management.

Serum Biochemical Parameters	Camels	Dromedaries	Llamas
Total Protein (mg/dL)	6.68 ± 0.25	6.08 ± 0.19	6.44 ± 0.31
Albumin (g/dL)	4.78 ± 0.08	4.64 ± 0.13	4.68 ± 0.10
Urea (mg/dL)	25.60 ± 8.29	61.60 ± 4.39	44.40 ± 19.98
ALP (U/L)	98.80 ± 42.66	130.80 ± 32.88	112.60 ± 32.63
GLU (mg/dL)	63.20 ± 32.40	14.40 ± 2.88	73.80 ± 11.25
GGT (U/L)	17.74 ± 6.25	5.42 ± 1.28	12.00 ± 8.22
GOT (U/L)	22.40 ± 6.73	31.60 ± 11.4	27.80 ± 4.17
GPT (U/L)	90.60 ± 42.15	69.60 ± 6.58	64.40 ± 26.33
Total bilirubin (mg/dL)	0.18 ± 0.04	0.10 ± 0.07	0.14 ± 0.08
Total cholesterol (mg/dL)	25.60 ± 6.54	31.80 ± 4.97	29.00 ± 7.59
Triglycerides (mg/dL)	13.00 ± 5.00	19.80 ± 11.9	19.00 ± 11.31
Ca (mg/dL)	9.18 ± 1.23	9.84 ± 0.15	9.68 ± 0.37
P (mg/dL	6.88 ± 4.44	12.60 ± 1.52	9.90 ± 3.53
CPK (U/L)	215.20 ± 57.65	146.60 ± 34.61	133.00 ± 5.59
Creatinine (mg/dL)	2.56 ± 0.60	1.54 ± 0.11	2.10 ± 0.67

## Data Availability

The data presented in this study are available on request from the corresponding author due to privacy reason.
